# A Rapid Cost Modeling Tool for Evaluating and Improving Public Health Supply Chain Designs

**DOI:** 10.9745/GHSP-D-20-00227

**Published:** 2020-12-23

**Authors:** Michael Krautmann, Mariam Zameer, Dorothy Thomas, Nora Phillips-White, Ana Costache, Pascale R. Leroueil

**Affiliations:** a William Davidson Institute, University of Michigan, Ann Arbor, MI, USA.; b VillageReach, Seattle, WA, USA.

## Abstract

Effective and efficient health supply chains play a vital role in achieving health outcomes by ensuring supplies are available for people to access quality health services. However, supplying health commodities to service delivery points is complex and costly in many low- and middle-income countries. Thus, governments and partner organizations are often interested in understanding how to design their health supply chains more cost efficiently.

Several modeling tools exist in the public and private market that can help assess supply chain efficiency and identify supply chain design improvements. These tools are generally capable of providing users with very precise cost estimates, but they often use proprietary software and require detailed data inputs. This can result in a somewhat lengthy and expensive analysis process, which may be prohibitive for many decision makers, especially in the early stages of a supply chain design process. For many use cases, such as advocacy, informing workshop and technical meetings, and narrowing down initial design options, decision makers may often be willing to trade some detail and accuracy in exchange for quicker and lower-cost analysis results. To our knowledge, there are no publicly available tools focused on generating quick, high-level estimates of the cost and efficiency of different supply chain designs.

To address this gap, we designed and tested an Excel-based Rapid Supply Chain Modeling (RSCM) Tool. Our assessment indicated that, despite requiring significantly less data, the RSCM Tool can generate cost estimates that are similar to other common analysis and modeling methods. Furthermore, to better understand how the RSCM Tool aligns with real-world processes and decision-making timelines, we used it to inform an ongoing immunization supply chain redesign in Angola. For the use cases described above we believe that the RSCM Tool addresses an important need for quicker and less expensive ways to identify more cost-efficient supply chain designs.

## Abstract (precis)

The Rapid Supply Chain Modeling Tool enables health system leaders to quickly estimate and compare the cost impact of potential supply chain design improvements in situations where time and budget do not allow for more in-depth modeling approaches.

## Teaser

**Key Findings:**

- The Rapid Supply Chain Modeling (RSCM) Tool addresses a need for more rapid and flexible ways to model the cost impact of changes to a country’s supply chain design or context.
- We compared the RSCM Tool against existing cost modeling tools and found it capable of producing similar results across a wide range of countries and supply chain designs.
- The ideal user for the RSCM Tool is a technical officer familiar with Excel and supply chain concepts; the outputs can inform both technical discussions and high-level policy decisions.

**Key Implications:**

Health system leaders and their technical teams should consider using the RSCM Tool to streamline the beginning stages of a supply chain design initiative, particularly in the following use scenarios:

- Generating estimates of high-level impact to inform initial advocacy efforts
- Sustaining momentum from initial workshops by quickly addressing supply chain questions
- Narrowing down a wide range of initial supply chain design possibilities to help policy makers more quickly focus on the highest-impact design changes

## BACKGROUND

Supply chains are a key component of any well-functioning health system.^1^ For vaccines, medicines, and other health products to be effective at preventing and treating disease, they must be accessible to the people who need them, when and where they are needed. Health supply chains that can reliably deliver these products to the point of care are vital to ensuring access to quality health care and to achieving positive health outcomes.^2^ ^–^ ^4^

However, in many low- and middle-income countries (LMICs), health supply chains fail to ensure consistent availability of critical health products.^5^ According to the most recent World Medicines Report, a typical public clinic in sub-Saharan Africa averaged only 57% availability of its required essential medicines, and nearly 25% of all LMIC patients were regularly unable to access medicines needed for treatment.^6^ In addition, global vaccination coverage has plateaued at 80%–85% since 2010, and supply chain inefficiencies are considered a significant driver.^7^ ^,^ ^8^ These same supply chains are also expensive to operate, requiring millions of dollars of annual funding to supply thousands of public health facilities throughout a given country.^9^ ^,^ ^10^ For these reasons, improving health supply chain efficiency and effectiveness is a key objective for donor agencies, governments, and other health care stakeholders.^11^ ^–^ ^13^

One important pathway to achieving this objective is restructuring and improving a supply chain’s design (i.e., the overarching strategy for organizing a supply chain network and its human resources, technologies, and processes). Recent studies have demonstrated that improving a supply chain’s design can lead to more cost-efficient delivery and better product availability in health facilities.^14^ ^–^ ^18^ Thus, donor agencies like Gavi, the Vaccine Alliance, have explicitly incorporated supply chain design into their supply chain strategies,^19^ and country governments are prioritizing supply chain redesign and strategy development activities in their national health plans.^20^ ^,^ ^21^

The task of analyzing and identifying an improved supply chain design can often be challenging for a couple of reasons. First, detailed supply chain data (e.g., operating costs, product demand, facility locations) are often unavailable or time intensive to collect. Second, many existing tools used to collect and analyze such data are intended to provide a snapshot of the current system,^22^ whereas a design analysis requires flexible models that can predict the impact of large-scale changes to the supply chain. Although more sophisticated supply chain modeling and optimization tools do exist for this purpose, they typically require proprietary software and specialized modeling skills and/or consultants. In total, a supply chain review and redesign process using these current methods can require at least 3–6 months and US$250,000–US$500,000, according to recent estimates.^23^

The detailed and precise outputs from such tools are necessary in some circumstances, particularly at the final stages of a supply chain design process when the focus is on fine-tuning and implementing a specific plan. However, there are a broad range of other instances where the required time and cost are prohibitive. This could include situations like conducting initial advocacy for supply chain improvements, informing workshops and meeting discussions in real time, or narrowing down a wide range of improvement options in the early stages of a supply chain design process. In such cases, leaders from ministries of health and partner organization would likely trade some level of detail and accuracy in exchange for reducing the time and cost of analysis. To our knowledge, there are no publicly available modeling tools that are flexible enough to help decision makers evaluate the cost and efficiency of different supply chain designs, while also minimizing the need for data collection and specialized software skills.

In this article, we present the design and testing of a Rapid Supply Chain Modeling (RSCM) Tool aimed at addressing this gap. We describe key attributes of the RSCM Tool, validate its results against existing supply chain analyses, and explore how it can help inform a country’s supply chain redesign process.

To our knowledge, there are no publicly available modeling tools that are flexible enough to help decision makers evaluate the cost and efficiency of different supply chain designs while also minimizing the need for data collection and specialized software skills.

## METHODOLOGY: DESIGNING A TOOL FOR RAPID ANALYSIS USE CASES

The RSCM Tool is a quantitative, Excel-based model designed to quickly estimate costs and basic efficiency metrics for multiple supply chain design scenarios. It requires users to input information about the design and general characteristics of a country’s current supply chain, including: supply chain network information, such as land area and number of facilities; cost parameters, such as the cost of labor, fuel, or vehicles; and storage and distribution guidelines, such as inventory levels or frequency of delivery.

Using these inputs, the RSCM Tool models key operational supply chain activities and calculates several resulting output metrics, including: annual operating cost, disaggregated by tier and supply chain function (i.e., storage, transportation, and management); expected utilization of resources like vehicle and warehouse capacity; and operational statistics like kilometers traveled or volume delivered per facility.

Within the tool, those inputs and outputs worksheets can be replicated to create multiple supply chain scenarios, which can be compared under a main dashboard.

To address the identified need for quicker and more cost-effective decision-making tools, we made several design decisions that help the RSCM Tool maintain flexibility, quick setup time, and minimal data collection needs (Table 1).

**TABLE 1.** Key Modeling Tool Design Decisions for Facilitating Rapid Supply Chain Analyses


Simplifying modeling assumptions: Reducing data requirements and enable real-time calculation	Assumptions: All facilities at a given tier have the same demand quantity per order period Demand is the same for every order period and does not vary over time Facilities within a tier are evenly distributed throughout a given region and, thus, are the same average distance to their nearest re-supply point
Standardizing design levers: Providing flexibility to model diverse global health distribution strategies	Storage: At which levels do you hold and manage inventory? How much safety stock does each level hold, and how frequently is it replenished? Transportation: What types of vehicles are used to transport replenishment shipments? What type of distribution model is followed at each level (e.g., hub and spoke or multi-stop distribution loops), and are there any travel constraints (e.g., administrative boundaries)? Management: Who is responsible for performing key ordering, transport, and storage functions? What types of technology supports people at each level?
Proxying data and worksheets to fill gaps: Enabling quick estimation of missing data points	Supporting worksheets and datasets: A model for estimating immunization and/or reproductive health demand volumes and product value, by combining available demand planning methodologies with publicly available demographic and product data A general model for converting the number of units of a health product into a cubic-meter volume using historical product unit volume data Common commercial heuristics for estimating storage capacity of a warehouse based on its overall dimensions A database of typical costs for assets like vehicles, warehousing space, and cold chain equipment
Using Excel-based platform for broad accessibility

To address the need for quicker and more cost-effective decision-making tools, we designed the RSCM Tool to maintain flexibility, quick setup time, and minimal data collection needs.

### Simplifying Modeling Assumptions

Like most modeling tools, the RSCM Tool uses assumptions to strike a desired balance between simplicity and accuracy. Since our goal is to provide faster results by reducing overall data requirements, we ask the user to define a typical facility at each supply chain tier, rather than requiring detailed demand, location, and cost data for every facility.

While the results do not provide detailed outputs for individual facilities, we hypothesize that for high-level, system-wide design analyses, the RSCM Tool’s outputs will be reasonably similar to those of other common supply chain modeling tools. We test this hypothesis and quantify the effect of the tool’s assumptions in the validation section below.

### Standardized “Menu” of Design Levers

Most global health supply chains can be broken down into a relatively small set of “building block” design decisions in key functional areas like storage, transportation, and management. By incorporating these design levers along with common pre-set choices/values, a user can easily create and toggle between different distribution strategies for their supply chain network.

### Proxy Data and Worksheets to Address Data Gaps

Since supply chain and cost data are often scarce, we incorporated several supporting worksheets and proxy datasets to help users quickly estimate values for common data gaps, minimizing time required for data collection.^24^ ^–^ ^29^ Additionally, we compiled a set of complete input data templates, which are proxy data from existing cost analyses that are formatted to match the RSCM Tool’s structure. Instead of entering each input value individually, a user can “load” a preset template as a starting point for analysis, selectively overriding the proxy dataset where better data exists.

### Excel-Based Platform

Finally, we developed the RSCM tool in Microsoft Excel because it is the most widespread software that can meet the tool’s technical requirements and run easily on most computers. Government staff and implementing partners are often familiar with and comfortable using Excel in their daily work, such as for demand planning. For users who already have Excel, there is no additional cost to accessing the RSCM Tool. Additionally, the tool is functional offline, which is essential for areas with unreliable internet connectivity. This enables the tool to be easily and widely accessible to multiple stakeholders throughout a country, which would be less likely if it required proprietary software or license fees.

## VALIDATING THE RSCM TOOL’S METHODOLOGY

To test the validity of the methodology and assumptions described, we conducted an assessment to determine whether the outputs generated by the RSCM Tool were consistent with other established methods for measuring or estimating the costs of different supply chain designs.

### Validation Approach

Our general approach was to compile detailed datasets from recent supply chain costing and modeling analyses and replicate each analysis using the RSCM Tool. First, we built complete sets of data inputs for the RSCM Tool, compiling them from a variety of sources and vetting assumptions externally wherever possible. (The Discussion details the main challenges we faced in building these input datasets and how we addressed them.) Then, we compared the RSCM Tool’s cost estimates to the results of the original analyses.

With identical data inputs, we would expect any discrepancy in results to be driven by differences in the modeling approach and assumptions. For each comparison, we treated the existing analysis as a “reference” value and measured the RSCM Tool’s deviation from that value as an absolute percent error.

We performed this calculation at 3 levels of cost aggregation: (1) individual cost line items (e.g., fuel costs for transportation at health facilities); (2) total cost for each supply chain tier and cost category; and (3) total annual cost for the entire system. For each level of aggregation, we averaged individual error calculations together to obtain a mean absolute percent error (MAPE). A lower MAPE value implies a smaller difference between the reference and RSCM tool results.

We were able to obtain reference datasets from 6 recent cost and modeling analyses, covering 7 supply chain design scenarios (Table 2). We chose these reference analyses in part based on our ability to access underlying data since replicating the analyses as closely as possible required a more detailed breakdown of inputs and results (e.g., worker salary assumptions, specific vehicle types) than what is typically available in public reports. Additionally, we sought out analyses that were produced and vetted by country governments and partners and actually used to inform key stakeholder decisions. Even though these analyses also represent estimates of true supply chain costs, they are the best-established estimates available, and thus, serve as ideal reference values when validating the RSCM Tool.

**TABLE 2.** Reference Datasets From Cost and Modeling Analyses Used to Validate the Rapid Supply Chain Modeling Tool

**Study Location**	**Description**
Bolivia and Guatemala, 2018	Three supply chain costing studies led by ForoLAC (Foro Latinoamericano y del Caribe para el Aseguramiento de Insumos de Salud Reproductiva) that included all major health commodities, including vaccines: Tarija Department, Bolivia Quiché Department, Guatemala Alta Verapaz Department, Guatemala
Mozambique, 2015	Modeling analysis conducted by VillageReach for the national and provincial ministries of health, using the HERMES software platform (Highly Extensible Resource for Modeling Event-Driven Supply Chains) to assess 2 immunization supply chain design options for Manica Province in Mozambique (the baseline 4-tier design, and a direct delivery design that skipped 1 of the tiers).
Senegal, 2017	Modeling analysis in Senegal estimating the nationwide costs of operating the Informed Push Model strategy for delivering family planning and maternal-child health products.
Zimbabwe, 2015	Evaluation of the Zimbabwe Assisted Pull System strategy in Manicaland Province in Zimbabwe, which integrated commodity distribution for most health program areas (except vaccines).

Collectively, these analyses encompass a diverse set of current public health supply chains. They incorporate several health program areas and span a range of geographies across Africa and Latin America. They also cover several common supply chain designs, including ad hoc facility collection, “level-skipping” or “direct delivery” designs that bypass an administrative tier and a “mobile warehouse” design where facility inventory is periodically topped-up by visiting resupply vehicles.^30^

### Adjusting Data to Ensure Equivalent Comparisons

These 6 reference analyses were conducted by different organizations for different purposes; hence, they differ in methodological details like the scope of costs included, how costs are classified, and analysis method (e.g., simulation modeling vs. direct cost measurement at a sample of facilities). None of these methods is inherently better than another; each uses a set of data and assumptions that are tailored to its own unique context. However, due to these differences, we often needed to transform certain data inputs and outputs to ensure an equivalent comparison with the RSCM Tool.

Many of the input parameters required by the RSCM Tool lacked a directly comparable value in the reference analyses, requiring us to make several types of estimates and adjustments, including:

- **Aligning Level of Detail:** Many RSCM inputs were available in the reference datasets but were scoped or grouped differently. For example, the RSCM Tool handles vehicle costs like fuel, maintenance, and insurance individually, but some datasets use only an aggregate “total operating cost” rate, requiring us to estimate the breakdown of that rate into its subcomponents.
- **Inferring Input Values From Results:** With some datasets (especially ones that only had results available), we lacked explicit assumptions for required inputs like vehicle maintenance cost rates. However, in many cases, we were able to infer a value from data contained in the results, such as overall maintenance costs and distances traveled. While we would ordinarily avoid using the detailed reference datasets as sources for RSCM Tool inputs, we were comfortable doing so in situations where the input parameter: (1) was an objective, numeric value, and; (2) would likely be found elsewhere in ministry or partner financial records that would be accessible in a country-level application of the tool.
- **Identifying Proxies for Missing Data Inputs:** Some RSCM inputs simply were not available in a reference dataset, often because of a difference in methodology. For example, the RSCM Tool uses a road network circuity factor to help estimate distances between facilities. We often had to use Google Maps to develop a rough proxy for this parameter because many of the reference analyses measured actual distances between sample facilities.

Similarly, when comparing final outputs, in the following examples, we often had to adjust for differences among the reference analyses in how specific cost line items were calculated. For example:

- **Costing Unutilized Assets**: Some reference analyses and the RSCM Tool track all assets that are owned by a supply chain (e.g., vehicles or storage space), while other analyses track only the fraction of those assets that are actively used. Both approaches are valid but result in different answers unless the assets are fully utilized. Thus, when comparing against this alternate approach, we scaled down the quantity of vehicles and storage in the RSCM Tool to eliminate any expected idle capacity.
- **Assigning “Ownership” of Costs** *:* The RSCM Tool assigns the cost of a supply chain activity to the location where that activity occurred. Certain analyses, however, assign costs to wherever the budget line for those costs is located. Those are not always the same locations (e.g., if health facility vehicle maintenance is funded out of a district budget).
- **Scoping Specific Cost Categories:** Reference analyses differed in the scope of costs that they were willing to consider. For example, several analyses omitted depreciation costs for health facility storage space, since the buildings are often owned by the government and require no rent or mortgage payment. Others chose to include a nominal storage space cost, since eventually that health facility building would need to be replaced.

### Validation Results

**FIGURE 1.**
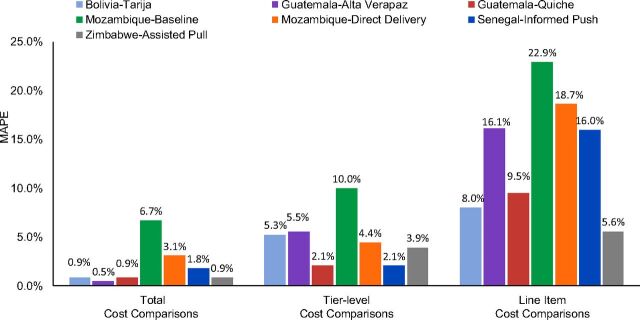
Comparison of Cost Estimates Between the Rapid Supply Chain Modeling Tool and Existing Reference Supply Chain Analyses Abbreviation: MAPE, mean absolute percent error.

Figure 1 shows the difference, measured in MAPE, between the RSCM Tool’s cost estimates and those of the 7 reference supply chain scenarios. MAPE values were lowest when comparing total supply chain operating costs between the RSCM estimates and the reference analyses. At this level, the only comparison to exceed a 4% MAPE value was the Mozambique baseline (6.7%). This implies that the modeling assumptions and simplifications described above generally have the smallest impact on high-level cost estimates. As the comparisons became more granular (e.g., comparing costs for an individual tier or cost line item), the differences became somewhat more pronounced.

We also saw a wide range of MAPE values across the different reference analyses. Some, such as the 2 Mozambique scenarios, saw relatively high MAPE values across all 3 levels of comparison. Others, such as Zimbabwe and the ForoLAC (Foro Latinoamericano y del Caribe para el Aseguramiento de Insumos de Salud Reproductiva) studies, were relatively low across the board. These differences appear to roughly correlate with the level of detail available in the underlying reference datasets. We discuss further implications of these results in the Discussion section.

## TESTING THE TOOL IN AN IMMUNIZATION SUPPLY CHAIN CONTEXT

We also wanted to test the usability of the tool to understand how it aligned with real-world processes and timelines for conducting supply chain design analyses. In January 2019, United Nations Children’s Fund (UNICEF), with the support of technical partners, worked with Angola’s Expanded Program on Immunization (EPI) to explore a review and redesign of its immunization supply chain (iSC). This engagement provided us with an opportunity to evaluate whether the RSCM Tool could be used quickly and easily to produce high-level estimates during a real-time supply chain redesign.

### Angola Immunization Supply Chain Context

**FIGURE 2.**
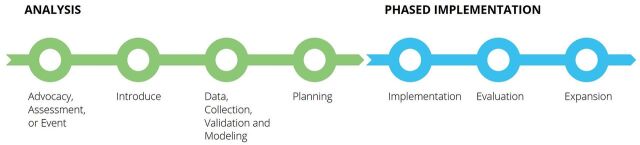
United Nation’s Children’s Fund System Design Approach Used to Review Angola’s Expanded Program on Immunization Supply Chain

Angola’s immunization supply chain consists of 4 tiers (national, province, municipality, and health facility) that align with the Ministry of Health’s administrative structure. Vaccines travel through national, provincial, and municipality stores on their way to 1,321 health facilities. By conducting a supply chain design review, UNICEF and EPI aimed to improve the efficiency of this iSC structure and understand how those improvements would impact deployment of resources like vehicles and cold chain equipment. UNICEF’s System Design Approach (Figure 2) provided an overarching framework for this design review process.^31^

We incorporated the RSCM Tool into the data collection, validation, and modeling stage of this process after an initial stakeholder workshop in May 2019 and conducted a preliminary modeling analysis while awaiting development of a more detailed optimization model. By providing quick interim results, our goals were to sustain stakeholder interest and momentum post-workshop, get government buy-in for the scenarios defined at the workshop, and streamline subsequent modeling analyses to focus on the most promising areas of improvement.

### Analysis of 4 Design Scenarios Using the RSCM Tool

We used the RSCM Tool to analyze the following 4 supply chain design scenarios that EPI representatives and partners identified at the initial stakeholder workshop.

1. A baseline scenario represented the current-state supply chain design, including the 4-tier structure, transportation strategy, and monthly resupply frequency.
2. An “ideal baseline” scenario, which maintained the current supply chain design but increased cold storage capacity to account for current shortages, represented the “true” cost to run the current design with added cold storage.
3. A reduced resupply frequency scenario lowered transport costs by switching from monthly to 2-month resupply cycles at the municipality level.
4. A level-skipping scenario bypassed the province level, with the national warehouse delivering supplies monthly to municipalities, resulting in a 3-tiered distribution structure.

The process for collecting data and analyzing these scenarios involved several key steps: (1) interviewing national-level EPI officials about key supply chain policies, (2) compiling and entering available data into an RSCM Tool data template, (3) identifying proxy data sources to fill any remaining data gaps, and (4) identifying how to model each design scenario in the RSCM Tool. In total, this process required approximately 3 weeks’ worth of personnel time, divided across 3 people. However, that time requirement could have been reduced significantly (30%–50% in our estimation) in a scenario where everyone working on the analysis was located together in Angola and fluent in Portuguese, the official language in Angola.

### RSCM Modeling Results

**FIGURE 3.**
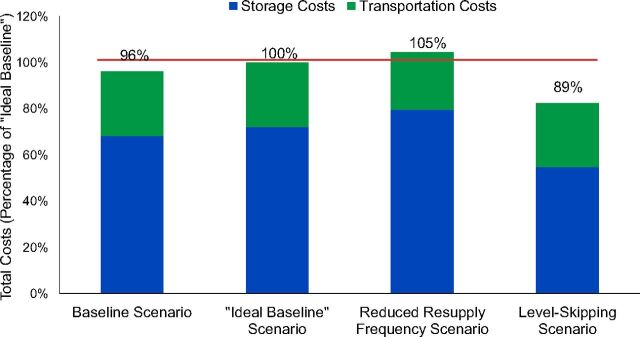
Change in Total Annual Operating Cost Estimates by Design Scenario for Angola’s Immunization Supply Chain, With Costs Shown as a Percentage of “Ideal Baseline” Costs

Total cost estimates from our analysis of these scenarios are shown in Figure 3.

For the analysis, we referenced the “ideal baseline” as 100% as it represents the “true” cost of running the supply chain; this reflects the added cost Angola would need to invest in the system regardless of any design changes. Based on the results, the level-skipping approach appears to be the most cost-efficient option, reducing annual costs by 7% over the current baseline and 11% over the ideal baseline. This difference translates to potential savings of several hundred thousand US dollars per year. Apart from the obvious reduction in storage costs, bypassing the provincial level also lowered overall transport costs by enabling more efficient transportation routes from national level to municipalities.

We presented these initial results to stakeholders from UNICEF’s Supply Division and the Angola country office in August 2019, and they will be using the results to get government buy-in for subsequent in-depth modeling. Testing the RSCM in Angola provided an opportunity to assess the RSCM Tool’s ability to quickly estimate results for stakeholders and provide guidance on which options are worth exploring in more depth.

## DISCUSSION

We tested the RSCM Tool in 2 ways: (1) by validating it against existing supply chain costing analyses, and (2) by using it to help inform an ongoing supply chain design review in Angola. We discuss the outcomes of those testing processes and what they mean for the usability and limitations of the RSCM Tool.

### Interpretation of the Validation Results

The validation comparisons shown in Figure 1 generally align with our expectations of what a high-level, rapid tool should be able to achieve. The RSCM Tool was very good at replicating total operating cost numbers from the reference analyses. Even at the tier- and category-specific level, estimates were often within ±5% of the reference analysis results.

The RSCM Tool adequately replicated total operating cost numbers from the reference analyses.

It is not surprising that the RSCM Tool and the reference analyses differ somewhat in their results, given the differences in their underlying modeling assumptions. For example, we assume identical demand and travel distances for all facilities within a given tier, but there is often variation among real-world facilities (e.g., large, accessible urban health facilities vs. small, remote rural health facilities). Those differences tend to average out over a large sample of facilities, but even at a national level, this assumption likely contributes to the MAPE values in Figure 1. The data adjustments we described in the Methodology section also likely contribute to these differences.

Our key question, then, is determining what constitutes an acceptable MAPE level for the comparisons shown in Figure 1. This is challenging because the definition of “acceptable” varies with the urgency and importance of the use case. Users who need answers very quickly or cheaply are likely to accept larger discrepancies than those who have more resources or a larger decision at stake. For this question, the literature on forecasting accuracy (where MAPE is an important metric) may provide the best guidance regarding what is generally considered acceptable. Landscape reviews of published forecasts provide numerous examples of both public and private organizations willing to make strategic decisions with error rates of 10%–20% or more.^32^ ^–^ ^34^ Thus, we assume that similar rates for the validation study (<10% MAPE at the total cost level, and <20% MAPE for individual line items), would be generally acceptable given the “rapid” use cases we are targeting. We presented this proposed threshold at several global health stakeholder consultations, workshops, and conferences, and received general approval from participants.^35^ ^,^ ^36^ However, as we suggest earlier, the user must ultimately define what level of accuracy is warranted.

Furthermore, like the RSCM Tool, the reference analyses from our validation are also estimates of true supply chain costs, based on other modeling tools and data analyses. Thus, they are also subject to similar accuracy challenges, including sample and observation bias, unreliable informants, and assumptions about scaling and interpreting data. We used these existing analyses as reference values because they represent the best data currently available, but they are likely not a perfect representation of true supply chain costs, and the MAPE values shown in Figure 1 should be interpreted with that in mind. Rather than being a true “accuracy” measure, the MAPE values represent evidence that, given similar inputs, the RSCM Tool can generate supply chain cost estimates that are comparable to other established methods.

### Lessons From an Immunization Supply Chain Design Context

Although Angola’s iSC system redesign is still in process, the RSCM Tool was able to generate helpful information for stakeholders:

- Overall estimated operating cost of the current supply chain
- The rough magnitude of cost savings that could result from implementing an improved iSC design
- The estimated cost of addressing current cold chain equipment shortages
- Evidence for the types of improvements (i.e., level skipping) that will likely have the largest financial impact

Having these data points early in the design process can help the EPI better prioritize the effort it puts into iSC design improvements (and compare against other supply chain or health system improvements). It can also inform specific requests for subsequent deep-dive analyses, such as identifying optimal modes of transport for a level-skipping solution or fine-tuning the regions where this solution should apply.

The RSCM Tool generally fared well with the data and modeling challenges we encountered during this analysis in Angola. Data visibility was an issue at municipality and health facility levels, but national-level government staff were able to describe the “typical” municipality and health facility easily. We were able to use proxy data and supporting worksheets to estimate other missing values (e.g., typical demand per facility, government salary rates, and average distances between storage facilities at each tier). We also developed an estimation method to capture the effect of supplemental immunization activities, which occur in Angola every 6 months and create a temporary 30% increase in throughput during those months. The RSCM Tool does not inherently consider month-to-month differences in demand volume, but we were able to use the tool’s storage/transport utilization estimates to add buffer capacity to handle those temporary demand spikes. In these ways, we were able to address substantial data visibility and modeling challenges and produce results in a relatively quick timeframe.

Most importantly, the process of working with external stakeholders to conduct this analysis helped clarify 2 key lessons for enabling long-term external use of the RSCM Tool.

#### 1. Users Must Be Familiar With Data Analysis and Supply Chain Concepts

The tool, while designed for ease of use, is not a substitute for data analysis and supply chain knowledge. We have included various features to maximize usability of the tool (e.g., user guides, detailed interpretation notes, formatting to highlight errors and omissions), but we cannot predict all the possible analysis situations and challenges that future users might encounter. The user must be capable of making informed judgments about how to utilize imperfect data sources and align them with the RSCM tool’s structure, and how to handle novel modeling situations. Thus, a technical or logistics officer or someone familiar with assessing supply chain operating costs is likely to be the ideal long-term user of the RSCM Tool within a government or partner organization.

#### 2. Adopting the Tool May Require Sustained Engagement

Transferring long-term use of the RSCM Tool to an external organization may still require sustained engagement, at least while the tool is relatively new and unknown. Our Angola analysis succeeded in generating interest in the tool, but it was a proof-of-concept for many stakeholders. They needed to see that initial analysis conducted before they would consider using the tool themselves the next time around. Thus, while we designed the tool so that anyone with Excel can download it and understand how to use it, we recognize that in many cases effective dissemination will involve direct engagement with country and partner stakeholders over the course of multiple use cases and analyses.

### Limitations of the RSCM Tool Methodology

Although the RSCM Tool can cover a wide range of supply chain designs and contexts, in the following situations, the tool’s structure and assumptions are likely to create bias or error in the results.

In some supply chains a single “typical” facility may be difficult to define. This can occur for regions with unusual geographies, or with facilities highly concentrated in 1 area. It could also include supply chains with extremely variable demand over time or across facilities. These supply chains likely require more detailed data to model in the RSCM Tool, since built-in worksheets and proxy data may be less representative, and assumptions may change significantly between different scenarios.

Some supply chains utilize different strategies for different subregions or program areas. If supply chain designs are not consistent across products or facilities (e.g., a region that uses a different distribution strategy than the rest of the country), the RSCM Tool requires the user to create separate models for each design and then aggregate the results.

For small supply chain networks, a single outlier facility can greatly influence results. While the RSCM Tool can model very small regions (e.g., a district with 20 health facilities), there is a much greater risk that a single outlier facility could skew the results. Those outliers will tend to average out over a large enough sample, so our analyses thus far have typically modeled at least an entire province/region (generally more than 100 health facilities).

These limitations are an important consideration when deciding whether to use the RSCM Tool for a given analysis. If addressing the limitation is serious enough that it requires a large quantity of individual health facility data to resolve, then users should also consider other modeling tools that can utilize detailed facility-level data. However, if the limitation can be addressed with a relatively small fix (e.g., manually adjusting a few parameters or creating a few scenarios), then the RSCM Tool may still be suitable.

### Broadening Access to Supply Chain Design Analysis

Given the RSCM Tool’s characteristics and limitations, we can envision it being useful in a wide range of situations where health system leaders need quick, high-level supply chain cost and design insights but lack the resources or time for a comprehensive data collection and in-depth modeling analysis.

We can envision the RSCM Tool being useful in situations where health system leaders need quick, high-level supply chain cost and design insights but lack the resources or time for a comprehensive data collection and in-depth modeling analysis.

#### Advocating for Supply Chain Initiatives

Building initial political support for supply chain improvements often requires advocates to demonstrate the potential cost and impact of those improvements but until that support exists, resources are often unavailable to conduct in-depth and costly analysis. Being able to quickly generate high-level, country-specific data can help advocates more effectively build initial political support.

#### Prioritizing Health Investment Decisions

Health system leaders must allocate funding across numerous initiatives to maximize health impact but cannot intensively analyze all potential options. High-level supply chain cost data can help leaders better compare supply chain improvements with other health investments.

#### Streamlining Traditional Supply Chain Redesign Processes

As our engagement in Angola demonstrated, the RSCM Tool can serve as a preliminary filter for more in-depth modeling analysis, quickly narrowing down a wide range of initial design possibilities. In this way the RSCM Tool can enable a more targeted use of complex, expensive modeling software by allowing to tools to focus only on the options with the most potential. Sequencing both tools in this fashion can facilitate a quicker and lower-cost supply chain redesign process that still yields sufficient detail where needed (e.g., for final budgeting and implementation planning).

#### Informing Real-Time Workshop/Meeting Discussions

When starting from a pre-existing data template, the RSCM Tool can generate scenario analyses in a matter of minutes or hours. This opens the possibility of conducting supply chain analyses in everyday government/donor meetings or addressing workshop questions in real-time.

#### Tailoring Funding Allocations

Many donor and government organizations allocate supply chain funding as a percentage of commodity value,^22^ which is a quick method but often uncorrelated with logistics costs. The RSCM Tool can provide quick estimates based on a more predictive attribute (volume) and tailored to a specific country context.

#### Validating Logistics Company Bids

Outsourcing supply chain activities is another situation where cost information is valuable but does not warrant its own extensive study. Having a readily available approximation of supply chain costs can help donors and governments more effectively negotiate with private logistics companies when they are bidding for services.

The use scenarios described in this article are not well-served by the existing landscape of supply chain tools, as they are typically diffuse, short-lived, and often too small or early stage to warrant a significant resource investment for analysis on their own. However, they are still important in building towards and sustaining supply chain outcomes. By providing stakeholders with rapid, data-driven insights in these types of diffuse situations, we can better initiate and sustain policy discussions about supply chain, more efficiently build consensus around the right types of solutions, and more effectively generate political will for larger-scale supply chain analyses and initiatives.

By providing stakeholders with rapid, data-driven insights, we can better initiate policy discussions about supply chain, more efficiently build consensus around solutions, and more effectively generate political will for supply chain analyses and initiatives.

## CONCLUSION

Identifying, costing, analyzing supply chain design improvements has traditionally been a highly time- and resource-intensive process. The RSCM Tool reduces these barriers, foregoing some degree of detail and accuracy to minimize data collection and enable quick turnaround of results. We tested and validated the RSCM Tool and found it capable of replicating high-level results of more traditional costing and modeling approaches. It also adapted well to existing country-level supply chain redesign processes, helping generate quick preliminary results that guide more in-depth modeling and decision making. We believe the RSCM Tool can help health system leaders make more timely and informed supply chain decisions, helping ensure efficient and reliable access to health products that are critical to improving health outcomes.

## Notes

**Peer Reviewed**

**First published online:** November 20, 2020

## References

[1] Management Sciences for Health (MSH). *MDS-3: Managing Access to Medicines and Health Technologies* . MSH; 2012. Accessed October 29, 2020. https://www.msh.org/sites/default/files/mds3-jan2014.pdf

[2] Seidman G , Atun R . Do changes to supply chains and procurement processes yield cost savings and improve availability of pharmaceuticals, vaccines or health products? A systematic review of evidence from low-income and middle-income countries. BMJ Glob Health. 2017;2(2):e000243. 10.1136/bmjgh-2016-000243. 28589028 PMC5435270

[3] World Health Organization (WHO); Bigdeli M , Peters DH , Wagner AK . *Medicines in Health Systems: Advancing Access, Affordability and Appropriate Use* . WHO; 2014. Accessed October 29, 2020. https://apps.who.int/iris/bitstream/handle/10665/179197/9789241507622_eng.pdf

[4] Donato S , Parry J , Roth S . *ADB Brief: Strong Supply Chains Transform Public Health* . Asian Development Bank; 2016. Accessed October 29, 2020. https://www.adb.org/sites/default/files/publication/214036/strong-supply-chains.pdf

[5] Yadav P . Health product supply chains in developing countries: diagnosis of the root causes of underperformance and an agenda for reform. Health Syst Reform. 2015;1(2):142–154. 10.4161/23288604.2014.968005. 31546312

[6] Wagner AK , Graves AJ , Reiss SK , LeCates R , Zhang F , Ross-Degnan D . Access to care and medicines, burden of health care expenditures, and risk protection: results from the World Health Survey. Health Policy. 2011;100(2-3):151–158. 10.1016/j.healthpol.2010.08.004. 20828854

[7] Glassman A , Oroxom R , Silverman R , Madan Keller J , Kenney C , Schnabel L . *Global Immunization and Gavi: Five Priorities for the Next Five Years* . Center for Global Development; 2019. Accessed October 29, 2020. https://www.cgdev.org/sites/default/files/global-immunization-and-gavi-five-priorities-next-five-years.pdf

[8] Mihigo RM , Okeibunor JC , O’Malley H , Masresha B , Mkanda P , Zawaira F . Investing in life saving vaccines to guarantee life of future generations in Africa. Vaccine. 2016;34(48):5827–5832. 10.1016/j.vaccine.2016.06.036. 27342915

[9] Rosen JE , Bancroft E , Hasselback L , Levin C , Mvundura M , Tien M . *Last Mile Costs of Public Health Supply Chains in Developing Countries: Recommendations for Inclusion in the United Nations OneHealth Model* . United States Agency for International Development, DELIVER PROJECT; 2012. https://www.psmtoolbox.org/wp-content/uploads/2017/11/LastMileCost.pdf

[10] Shretta R , Johnson B , Smith L , et al . Costing the supply chain for delivery of ACT and RDTs in the public sector in Benin and Kenya. Malar J. 2015;14(1):57. 10.1186/s12936-014-0530-1. 25652315 PMC4341244

[11] United States Agency for International Development (USAID). *USAID Vision for Health Systems Strengthening 2015–2019* . USAID; 2015. Accessed October 28, 2020. https://www.usaid.gov/sites/default/files/documents/1864/HSS-Vision.pdf.

[12] The Global Fund. Message from the Executive Director-supply chain processes. Published April 28, 2017. Accessed February 6, 2020. https://www.theglobalfund.org/en/oig/updates/2017-04-28-message-from-the-executive-director-supply-chain-processes/

[13] Bill and Melinda Gates Foundation. Health systems strengthening: ensuring effective health supply chains. Published March 7, 2017. Accessed October 28, 2020. https://gcgh.grandchallenges.org/challenge/health-systems-strengthening-ensuring-effective-health-supply-chains-round-19

[14] Vledder M , Friedman J , Sjöblom M , Brown T , Yadav P . Improving supply chain for essential drugs in low-income countries: results from a large scale randomized experiment in Zambia. Health Syst Reform. 2019;5(2):158–177. 10.1080/23288604.2019.1596050. 31194645

[15] World Health Organization (WHO). *Evidence Brief: System Design Approach to Improve the Immunization Supply Chain* . WHO; 2018. Accessed October 28, 2020. https://apps.who.int/iris/bitstream/handle/10665/272853/WHO-IVB-18.01-eng.pdf

[16] Lee BY , Haidari LA , Prosser W , et al . Re-designing the Mozambique vaccine supply chain to improve access to vaccines. Vaccine. 2016;34(41):4998–5004. 10.1016/j.vaccine.2016.08.036. 27576077 PMC5547748

[17] Brown ST , Schreiber B , Cakouros BE , et al . The benefits of redesigning Benin’s vaccine supply chain. Vaccine. 2014;32(32):4097–4103. 10.1016/j.vaccine.2014.04.090. 24814550

[18] Sarley D , Mahmud M , Idris J , et al . Transforming vaccines supply chains in Nigeria. Vaccine. 2017;35(17):2167–2174. 10.1016/j.vaccine.2016.11.068. 28364926

[19] Gavi The Vaccine Alliance. *Strengthening the Immunisation Supply Chain* . Gavi; 2016. Accessed October 29, 2020. https://peoplethatdeliver.org/ptd/download/file/fid/424

[20] United States Agency for International Development (USAID), DELIVER PROJECT. *USAID | DELIVER PROJECT Final Country Report: Tanzania* . USAID, DELIVER PROJECT; 2016. https://deliver.jsi.com/wp-content/uploads/2016/12/FinaCounRepo_TZ.pdf

[21] Republic of Ghana Ministry of Health (MOH). *Health Commodity Supply Chain Master Plan* . MOH; 2012. Accessed October 29, 2020. http://iaphl.org/wp-content/uploads/2016/05/GhanaSCM-2013.pdf

[22] McCord J , Tien M , Sarley D . *Guide to Public Health Supply Chain Costing: A Basic Methodology* . United States Agency for International Development, DELIVER PROJECT; 2013. Accessed October 29, 2020. https://publications.jsi.com/JSIInternet/Inc/Common/_download_pub.cfm?id=18156&lid=3

[23] United Nations Children’s Fund (UNICEF). *System Design Summit Final Report* . UNICEF; 2017.

[24] World Health Organization (WHO). Effective Vaccine Management (EVM) Assessment Tool. Accessed October 29, 2020. https://www.who.int/immunization/programmes_systems/supply_chain/evm/en/index3.html

[25] United Nations Population Division. World population prospects 2019. Published 2019. Accessed October 28, 2020. https://population.un.org/wpp/Download/Standard/Population/

[26] United Nations Population Fund (UNFPA). Contraceptive price indicators for the year 2017. Published 2018. Accessed October 28, 2020. https://www.unfpa.org/resources/contraceptive-price-indicator-year-2017

[27] United States Agency for International Development (USAID). Couple years of protection (CYP) conversion factors. Updated June 2, 2019. Accessed October 28, 2020. https://www.usaid.gov/global-health/health-areas/family-planning/couple-years-protection-cyp

[28] The Demographic and Health Survey Program STATcompiler. Accessed October 28, 2020. http://www.statcompiler.com

[29] United States Agency for International Development, DELIVER PROJECT. Master Listing of Product Volumes and Weights. 2008.

[30] Daff BM , Seck C , Belkhayat H , Sutton P . Informed push distribution of contraceptives in Senegal reduces stockouts and improves quality of family planning services. Glob Health Sci Pract. 2014;2(2):245–252. 10.9745/GHSP-D-13-00171. 25276582 PMC4168620

[31] United Nations Children’s Fund (UNICEF). System design approach. Accessed October 28, 2020. https://www.technet-21.org/iscstrengthening/en/system-design-approach

[32] E2Open. *2018 Forecasting and Inventory Benchmark Study* . E2Open; 2018.

[33] Nicolaisen MS , Driscoll PA . *Ex-post* evaluations of demand forecast accuracy: a literature review. Transp Rev. 2014;34(4):540–557. 10.1080/01441647.2014.926428

[34] Başar MS , Küçükönder H . Measuring the correlation between commercial and economic states of countries (B2G relations) and the E-Government Readiness Index by using neural networks. Open J Business Management. 2014;02(02):110–115. 10.4236/ojbm.2014.22014

[35] Krautmann M , Thomas D . Improving health supply chain design efficiency through rapid and flexible cost modeling. Paper presented at: Global Health Supply Chain Summit; November 28, 2018; Lusaka, Zambia. http://ghscs.com/wp-content/uploads/2018/12/1.3Improving-health-SC-through-rapid-cost-modeling82.pdf

[36] Krautmann M , Thomas D . A rapid modeling tool to improve reproductive health supply chain efficiency. Paper presented at: Reproductive Health Supplies Coalition- General Membership Meeting; March 27, 2019; Kathmandu, Nepal. https://www.rhsupplies.org/gmm2019/schedule.php#expo

